# Comment on: Global surgery education in Europe: a landscape analysis

**DOI:** 10.1093/bjsopen/zrac037

**Published:** 2022-03-08

**Authors:** Soham Bandyopadhyay

**Affiliations:** Oxford University Global Surgery Group, Nuffield Department of Surgical Sciences, Medical Sciences Division, University of Oxford, Oxford, UK

*Dear Editor*

I read the recent article by Velin *et al*. with interest^1^. They conducted a landscape analysis and arrived at the conclusion that only 16 universities had a global surgery centre. However, the results are at odds with the meaning of global surgery and possibly the reality of the situation.

It is critical when discussing global surgery, authors first become more familiar with the definition of global surgery: ‘an area of study, research, practice and advocacy seeking to improve health outcomes and achieve health equity for all people who need surgical and anaesthesia care’. The paramount point here is that global surgery is a field aiming to address inequity in surgical care, not just internationally, but nationally and locally as well. It is a field that considers all people: individuals in high-income countries (HICs) and low- and middle-income countries (LMICs). Global surgery being misinterpreted as a field focused on LMICs has led to numerous issues: previous reviews on global surgery led by junior researchers in HICs erroneously narrowing their focus to work conducted in LMICs, which has necessitated updates to be made to the existing body of literature; resources being diverted away from under-served populations in HICs; and power asymmetries between HICs and LMICs to name but a few.

These above reasons make it highly unlikely that only 16 medical schools across Europe have a global surgery centre. Most healthcare institutes have a strong focus on improving health outcomes for patients, including surgical patients. However, taking the authors’ narrower lens of global surgery, these numbers still do not stack up. The authors claim that there are no centres involved in global surgery in France, Spain, and Germany. However, this is not the case. The Centre Hospitalier Universitaire de Grenoble-Alpes in France is well known for its work with French-speaking LMICs, the Center for Surgical Studies at the Universidad Complutense in Spain has international surgical initiatives, and a collection of surgeons from various institutes in Germany lead the Deutsche Gesellschaft für Globale und Tropenchirurgie. The fact that these centres were missed highlights a flaw in the author’s landscape analysis, and the need for involving local stakeholders in the study design when conducting international studies.

Having said that the authors final remark of a need for expansion of global surgery education in Europe to meet the large interest among trainees and strengthen the role of European stakeholders in the global surgery discourse is well warranted.

**Author contributions:** This manuscript was conceived and written by S.B.

## References

[1] Velin L, Panayi AC, Lebbe I, Koehl E, Willemse G, Vervoort D. Global surgery education in Europe: a landscape analysis. BJS Open 2022;6:zrac001 Available from: https://academic.oup.com/bjsopen/article/6/1/zrac001/6526449 10.1093/bjsopen/zrac001 PMC8830757 35143626

